# Identification of novel bacterial biomarkers to detect bird scavenging by invasive rats

**DOI:** 10.1002/ece3.7171

**Published:** 2021-01-19

**Authors:** Carly R. Muletz‐Wolz, Erin Wilson Rankin, Sarah McGrath‐Blaser, Madhvi Venkatraman, Jesús E. Maldonado, Daniel S. Gruner, Robert C. Fleischer

**Affiliations:** ^1^ Center for Conservation Genomics Smithsonian Conservation Biology Institute National Zoological Park Washington DC USA; ^2^ Department of Entomology University of Maryland College Park MD USA; ^3^ Department of Entomology University of California Riverside CA USA; ^4^ Department of Biology University of Florida Gainesville FL USA

**Keywords:** biomarkers, decay, forensics, genomics, Hawaii, microbiome, noninvasive, predation, scavenging

## Abstract

Rapid advances in genomic tools for use in ecological contexts and non‐model systems allow unprecedented insight into interactions that occur beyond direct observation. We developed an approach that couples microbial forensics with molecular dietary analysis to identify species interactions and scavenging by invasive rats on native and introduced birds in Hawaii. First, we characterized bacterial signatures of bird carcass decay by conducting 16S rRNA high‐throughput sequencing on chicken (*Gallus gallus domesticus*) tissues collected over an 11‐day decomposition study in natural Hawaiian habitats. Second, we determined if field‐collected invasive black rats (*Rattus rattus*; *n* = 51, stomach and fecal samples) had consumed birds using molecular diet analysis with two independent PCR assays (mitochondrial Cytochrome Oxidase I and Cytochrome b genes) and Sanger sequencing. Third, we characterized the gut microbiome of the same rats using 16S rRNA high‐throughput sequencing and identified 15 bacterial taxa that were (a) detected only in rats that consumed birds (*n* = 20/51) and (b) were indicative of decaying tissue in the chicken decomposition experiment. We found that 18% of rats (*n* = 9/51) likely consumed birds as carrion by the presence of bacterial biomarkers of decayed tissue in their gut microbiome. One species of native bird (*Myadestes obscurus*) and three introduced bird species (*Lophura leucomelanos*, *Meleagris gallopavo*, *Zosterops japonicus*) were detected in the rats’ diets, with individuals from these species (except *L. nycthemera*) likely consumed through scavenging. Bacterial biomarkers of bird carcass decay can persist through rat digestion and may serve as biomarkers of scavenging. Our approach can be used to reveal trophic interactions that are challenging to measure through direct observation.

## Graphical abstract

We developed a novel three‐part approach using molecular dietary analysis and microbial forensics to reveal species interactions and scavenging by invasive rats on native and introduced birds in Hawaii. Our forensic genomic tool for use in ecological contexts is valuable for (a) detecting diet items in stomach and fecal samples, and (b) determining decomposition status of prey. Our method properly calibrated and replicated has great potential to detect and quantify species interactions that are remote, rare, and challenging to measure.

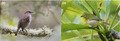

## 1 INTRODUCTION

Interactions among species impact biodiversity patterns, ecosystem responses to change, and ecosystem services (Tylianakis et al., 2008). The classic approaches for measuring species interactions involve direct and indirect observation, experimental manipulations of species or functional guilds, and predator stomach content analysis (Novak & Wootton, 2010; Paine, 1980; Ruffino et al., 2015). However, trophic interactions may be cryptic or rare and are often logistically challenging to measure. An integrated battery of techniques is often necessary to quantify predation by consumers, resolve trophic links, and to parameterize food web network models (Birkhofer et al., 2017; Carreon‐Martinez & Heath, 2010; Traugott et al., 2013). To date, these methods do not provide a solution for determining if a consumer is intaking a diet item as prey through predation or as carrion through scavenging. Ecologists often use indirect approaches (e.g., molecular or isotopic analyses) to identify that a food item was consumed, and the detection of a food item is generally classified as a predation event. Yet, scavenging may be involved in up to 45% of food web links and represents a substantial form of energy transfer between trophic levels that is unique from predation (Wilson & Wolkovich, 2011). For instance, 124‐fold more energy can be transferred per scavenging link than per predation link (Wilson & Wolkovich, 2011). Underestimating the role of scavenging in food webs likely impacts assessments of predator–prey interactions, energy flow, and important foodweb metrics. Likewise, the distinction between predation and scavenging has consequences for population and community dynamics (Moleon et al., 2014). Forensic genomics may be a useful technique to differentiate predation and scavenging to aid in our understanding of species interactions and food web ecology.

Forensic genomics used in an ecological context is an increasingly valuable tool for (a) detecting and identifying consumed prey in predator diet samples (invasively or noninvasively collected) and (b) determining rates of decomposition of an animal carcass, among others. First, molecular analyses of fecal material or gastrointestinal (GI) samples represent a technique to study diet and elucidate trophic interactions (McInnes et al., 2017; Rytkonen et al., 2019; Zarzoso‐Lacoste et al., 2016). These methods are based on PCR amplification of targeted diet items, using universal or species‐specific primer pairs, in samples collected from the consumer. DNA sequencing is then performed on the amplified PCR products either through Sanger sequencing (Zarzoso‐Lacoste et al., 2016) or high‐throughput sequencing (Rytkonen et al., 2019) and the resulting DNA sequences are compared to customized and/or public DNA reference databases (e.g., BOLD, NCBI GenBank) to identify the diet item. Second, molecular analyses of microbial community change over carcass decomposition time represents a unique way to study time since death (Belk et al., 2018; Metcalf et al., 2016; Pechal et al., 2014). This method is based on PCR amplification with universal primer pairs to target microbiota at domain‐level scales (e.g., bacteria, fungi) to identify microbial biomarkers associated with different time periods of decomposition. Microbial succession of particular taxa is often predictable across soil types, seasons and host species (Belk et al., 2018; Metcalf et al., 2016). Uniting these molecular methods can provide important food web insight into what omnivores/carnivores are consuming as diet items and whether particular diet items were likely consumed as prey through predation or carrion through scavenging. Quantifying rates of predation versus scavenging can provide novel insights into food web ecology and foster a more integrated understanding of how complex web dynamics interact (e.g., how prey or scavenging affects invasive population size, influences predation on vulnerable prey or affects food web stability; Moleon et al., 2014; Wolkovich et al., 2014; Zou et al., 2016).

Invasive rats have profound and wide‐ranging effects on island ecosystems and food webs (Clark, 1982; Shiels et al., 2014; Towns et al., 2006). For example, in Hawaii the invasive black rat (*Rattus rattus*) consumes a diversity of foods including seeds, fruits, arthropods, carrion, bird eggs and nestlings (Amarasekare, 1993; Cole et al., 2000; Levy, 2003). Over millions of years, Hawaiian island ecosystems have evolved in the absence of any functional analog to rats, with bats as the only native volant terrestrial mammal (Percy et al., 2008; Price & Clague, 2002). Thus, invasive black rats with their broad diet spanning the green and detrital food webs (i.e., multi‐channel omnivory: [Wolkovich et al., 2014]) may directly and indirectly impact key processes within invaded ecosystems.

**FIGURE 1:**
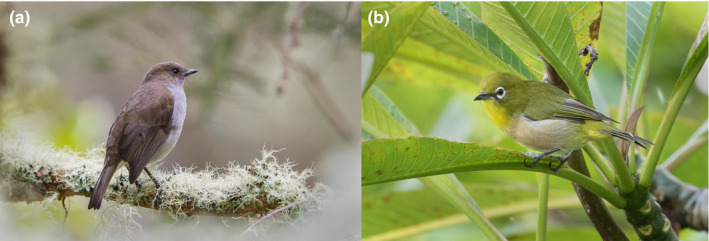
Invasive black rats (*Rattus rattus*) can directly and indirectly affect the ecology of native and introduced birds in Hawaii, such as the (a) native ʻōmaʻo (*Myadestes obscurus*) and the (b) introduced Japanese white‐eye (*Zosterops japonicus*) through modification of food web links. Photo credit: Jack Jeffrey

Invasive black rats can directly and indirectly impact the ecology and food web links for native and introduced bird species in Hawaii (Figure 1). Estimates of nest predation by black rats in Hawaii have ranged from high (e.g., 87% [Stone et al., 1984] to extremely low (e.g., 4% [Amarasekare, 1993]), indicating that black rats can have direct negative effects on reproductive success of native birds via predation, but this may vary by location. Rats can also have indirect effects on native and introduced birds by altering their foraging behavior and vertical habitat use, in turn impacting arthropod community biomass in the upper canopy (Wilson Rankin et al., 2018). Determining if invasive rats are preying upon birds, consuming birds as carrion, or not consuming birds will help ascertain the extent of the direct and indirect effects the rats are having on bird communities. If rats are directly consuming eggs, nestlings, fledglings or healthy adult birds, then such consumption could have a devastating direct effect on bird populations. If rats are consuming birds as carrion or rarely consuming birds in general, then the direct impact of rats on bird populations will be little to none (Fukami et al., 2006), although negative indirect impacts are still likely to occur (Wilson Rankin et al., 2018). Quantifying interactions among native and introduced species is critical to predicting the long‐term impacts of biological invasions and to understanding mechanisms for coexistence in these modified communities.

**FIGURE 2:**
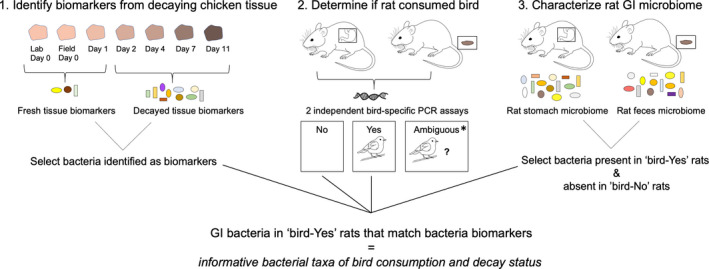
Conceptual figure of our three‐part approach to identify and validate informative bacterial biomarkers of bird consumption and carcass decay status (that are not part of the native rat GI microbiome). Asterisk indicates the potential to use informative bacterial taxa to resolve ambiguous status as to whether the rat consumed a bird or not

We developed a novel three‐part approach to identify potentially informative biomarkers of diet item decay status, determine if invasive black rats (*R. rattus*; GI samples) consumed birds, and to determine if the biomarkers could distinguish bird predation from bird scavenging (i.e., carrion consumption; Figure 2). First, we identified bacterial taxa that were associated with either fresh or decaying chicken tissue. Second, we determined whether rats had consumed birds using PCR‐based diet analysis. Third, we identified bacterial taxa that were both (a) detected only in rats that had consumed birds and (b) were indicative of fresh or decaying tissue in the experimental chicken model. We collected both rat stomachs and rat feces to validate that rat fecal samples, both less invasive and time‐intensive, would provide similar biological conclusions as stomach samples. We present evidence that bacterial biomarkers, linked to the successional stage of bird carcass decay, can persist as biomarkers through digestion to be measurable as biomarkers in both rat stomachs and noninvasive fecal material. Properly replicated through time and space and calibrated for use in a local system, such forensic genomic tools can detect and quantify species interactions that are remote, rare, and highly challenging to measure.

## 2 METHODS

### 2.1 Study location

We conducted a chicken (*Gallus gallus domesticus*) tissue decomposition study and collected black rat (*Rattus rattus*) fecal and stomach samples from rat traps in forest fragments, locally known as “kīpuka”. Hundreds of kīpuka were created by historical lava flows in 1855 and 1881 (Vaughn et al., 2014). The kīpuka we sampled are located on the NE slope of Mauna Loa Volcano in the Upper Waiakea Forest Reserve on the Island of Hawaii (19°40′N 155°20′W, 1,470–1,790 m elevation). These well‐replicated fragments vary in size, yet they share the same soils, origin, and a single dominant forest canopy species, ‘ōhi‘a lehua (*Metrosideros polymorpha*). Both native and non‐native animals inhabit these forests (Gruner, 2004), including birds endemic and introduced (Table S1) to Hawaii (Flaspohler et al., 2010). Hawaii has only one native terrestrial mammal *Aeorestes semotus* (Hawaiian hoary bat). All other terrestrial mammals present in the study system are non‐native, including *Rattus exulans* (Polynesian rat), *Rattus norvegicus* (Norway rat), *Mus musculus* (house mouse), *Herpestes javanicus* (Asian mongoose) and the most commonly encountered, *Rattus rattus* (black rat). Of these carnivorous and omnivorous species, only *R. rattus* demonstrably climbs trees and forages in forest canopies with regularity (Shiels et al., 2014; Vanderwerf, 2012; Wilson Rankin et al., 2018).

### 2.2 Identify bacterial biomarkers from decaying chicken tissue

To quantify microbial decay of bird tissue, we sampled decomposing chicken tissue at day 0 in the lab, and days 0, 1, 2, 4, 7, and 11 in the field at 10 locations (Table S2). First, we obtained a whole chicken carcass labeled antibiotic free from a grocery store (KTA Super Stores, Hilo HI). We split the chicken breast tissue into 70 equivalent samples weighing approximately 6 g on a sterile work surface in the laboratory and surface sterilized the chicken samples with 10% bleach. We created 10 sets of seven tissues samples and randomly assigned each set of samples to one of ten locations. Prior to leaving the lab, we immediately collected one sample from each assigned location and those served as the lab control samples (Day 0 lab; *n* = 10). In the field, in two separate kīpuka, we placed the remaining six samples at each of ten specified forested locations. These kīpuka were a subset of the larger study on the interactive effects of predation and ecosystem size on kīpuka food webs (spanning 34 kīpuka within a ~35 km^2^ range; Knowlton et al., 2017; Wilson Rankin et al., 2018). The two kīpuka for this chicken decomposition study were chosen as easily accessible, of intermediate size (3.19 and 2.77 ha for K18 and K19 respectively) and had rats present (rat removal controls in larger study). We chose to do the decomposition in the field to determine bacteria as biomarkers for decomposition in the same habitat as where the rats and birds occur. We placed each chicken tissue sample on a sterile piece of tin foil in a cleaned, closed rat trap (Havahart^®^) secured to the ground using clean, new tent stakes. Samples were placed in traps to avoid loss of experimental units during the decomposition experiment by rats. No ground scavenging insects were observed at the field sites, nor any evidence of such scavenging of tissue pieces. Immediately after placing the samples in the trap, we collected one sample from each location and those served as the field control samples (Day 0 field; *n* = 10). The remaining samples were collected during subsequent visits at Days 1, 2, 4, 7, and 11. We wore a fresh pair of sterile nylon gloves to collect each sample and placed it into 2 ml tubes in Queens College Buffer (20% DMSO, 0.25 M EDTA, 100 mM Tris, pH 7.5, saturated with NaCl; Amos et al., 1992). All vials (*N* = 70) were shipped to the Center for Conservation Genomics (CCG; National Zoological Park, Washington, DC) and stored in a −20°C freezer until analysis.

We conducted 454 pyrosequencing of 16S rRNA gene amplicons to characterize bacterial communities of the decomposing chicken tissue samples. We cut the tissue sample into 5 mm × 5 mm sections using scissors and tweezers sterilized with bleach and ethanol between each sample. We extracted DNA from 70 chicken tissue samples using the BioSprint 96 DNA kit (Qiagen) following the manufacturer's protocol for tissue extractions with an overnight incubation in lysis buffer and included negative extraction controls. We followed previously published methods (Muletz Wolz et al., 2018) to amplify the V3‐V5 16S rRNA gene region with the universal gene primer set 515F and 939R and to conduct library preparation. Each 25‐μl PCR reaction consisted of 1.25 U of AmpliTaq Gold DNA Polymerase (Thermo Fisher), 2.5 μM MgCl, 200 nM dNTPs, 200 nM reverse fusion primer, 400 nM forward barcoded fusion primer and 3 μl DNA template. PCR conditions were 95°C for 7 m, followed by 30 cycles of 95°C for 45 s, 55°C for 30 s, 72°C for 45 s and a final extension (72°C for 7 m). We used Speed‐beads (in a PEG/NaCl buffer; Rohland & Reich, 2012) to clean post‐PCR products, and pooled samples equimolar based on Qubit (Invitrogen) DNA concentration values. We conducted high‐throughput sequencing of samples using one Roche 454 GS Junior run and one GS FLX + run. Negative extraction controls and negative PCR controls were run with each 454 sequencing run to monitor for and then remove from analyses potential contaminant bacteria introduced in the laboratory.

We used R (R‐Core‐Team, 2019) and ‘dada2’ package (Callahan et al., 2016) to process the 454 reads following default parameters for 454 data, and to generate amplicon sequence variants (ASVs). We filtered the data to only contain ASVs that occurred at least once in at least two samples (i.e., filtered singletons). Four of the 70 samples (from Lab Day 0 and Field Day 0 samples) did not yield any sequences and were not included in analyses. To identify ASVs associated with particular sampling days across sites, we merged samples by Day across the ten replicate sites (see results—no significant differences in microbiome composition among sites were observed). We identified ASVs that we termed ‘fresh tissue biomarkers’, defined as ASVs present in only Day 0 lab, Day 0 field, and/or Day 1. We identified ASVs that we termed ‘decayed tissue biomarkers’, defined as ASVs present only in Days 2, 4, 7, and/or 11.

### 2.3 Field methods for capturing rats

As part of a larger study on the interactive effects of predation and ecosystem size on kīpuka food webs (spanning 34 kīpuka within a ~35 km^2^ range), rats were removed from 16 kīpuka, while 18 other kīpuka served as control plots (Knowlton et al., 2017; Wilson Rankin et al., 2018). In rat removal kīpuka, Victor M326 Pro Rat snap traps were set out and baited with peanut butter or coconut to quickly and ethically kill rodents upon entry (Stanford IACUC, no. 1776). Freshly killed rats (*n* = 23 from 10 kīpuka in current study; Table S3) were dissected and their entire stomach was preserved in 100% ethanol in the field. All equipment was disinfected with 10% bleach between dissections. In control kīpuka, two feeding stations (Black Trakka™, Gotcha Traps Limited) per hectare were set out and baited with peanut butter. Pilot studies conducted in 2012 demonstrated that each trap night yields 4–15 fecal pellets. One fecal pellet was preserved in 100% ethanol and frozen until processing (*n* = 28 from 8 kīpuka in current study; Table S3).

### 2.4 Diet analysis to determine if rats consumed bird

We extracted DNA from rat fecal and stomach samples using the Qiagen QIAamp DNA stool mini kit with modifications from manufacturer's protocol following previously published methods (Eggert et al., 2005; Wilbert et al., 2015) to increase DNA yields from fecal samples. We included a negative extraction control with each set of extractions. We used two independent bird‐specific primer pairs targeting conserved regions of the mitochondrial Cytochrome Oxidase I (COI) and Cytochrome b (Cytb) genes to determine if rats had consumed bird.

We analyzed a total of 23 rat stomach samples and 28 rat fecal samples, which were collected from different individuals. We used BIRDF1/AWCintR2 primer pair, which targets a ~350 bp region in the COI gene (Zarzoso‐Lacoste et al., 2013). BIRDF1/AWCintR2 was selected as most relevant from a wider range of primers based on similar types of samples (Zarzoso‐Lacoste et al., 2013, 2016). We used CytbCorL (5′‐ACTGCGACAAAATCCCATTC‐3′) and CytbCor3 (5′‐GACTCCTCCTAGTTTATTTGGG‐3′), which targets a ~233 bp region of the Cytb gene and was designed to target corvid DNA, but also previously amplified babbler DNA (Renner et al., 2008). Each 25‐μl PCR reaction consisted of 1.25 U of AmpliTaq Gold DNA Polymerase (ThermoFisher), 1.5 μM MgCl, 200 nM dNTPs, 200 nM reverse primer, 200 nM forward primer, 20 ng/μl BSA and 3 μl DNA template. PCR conditions were 95°C for 10 m, followed by 40 cycles of 95°C for 15, 30 s at the annealing temperature (57°C for BirdF1/AwCintR2 and 51°C for CytbCorL/CytbCor3 respectively), 72°C for 45 s and a final extension (72°C for 5 m). Any PCR products within the appropriate size range visualized via gel electrophoresis were then cleaned using ExoSAP‐IT (United States Bio‐chemical), and Sanger sequenced from the forward primer direction using the BigDye Terminator Cycle Sequencing Kit (Applied Biosystems, Inc). The sequenced products were column filtered, dried down, rehydrated with 10 μl of HPLC purified formamide, and then analyzed on an Applied Biosystems 3130xl DNA Analyzer. Individual sequences were assembled and edited in Sequencher 5.1 (GeneCodes). We sequenced 45 putative bird positive samples from BIRDF1/AWCintR2 primer pair and 23 putative bird positive samples from CytbCorL/CytbCor3 primer pair.

Samples were assigned a category of ‘consumed bird’, ‘not consumed bird’, or ‘ambiguous’ based on PCR results and Sanger sequencing. Every sample was tested at least twice, once with the COI primer set and once with the Cytb primer set. We considered samples as ‘consumed bird’ if at least one of the bird primer sets produced a high‐quality sequence (>50% quality) from Sanger sequencing, and that sequence matched a bird species in GenBank that we would expect to detect in our study site (Table S1). We blasted individual sequences in NCBI using BLASTN 2.9.0+. When all top matches (e‐value < e−50) matched a bird species in our study sites (native or introduced) with high query coverage (>95%) we recorded that bird species as the bird species consumed by the rat. We considered a sample as ‘not consumed bird’ if both bird primer sets did not amplify DNA in either of the COI and Cytb PCR reactions. We replicated the PCRs for the ‘not consumed bird’ for each primer set a second time to reduce the probability of false negatives. The negative samples were all tested a total of four times (two replicates per primer pair). We considered a sample as ‘ambiguous’ if at least one bird primer set produced a PCR band of appropriate target bp size, but we were unable to get a high‐quality sequence matching a bird sequence from Sanger sequencing after replicate sequencing attempts. Ambiguous samples were either (a) repeatedly low‐quality sequences, (b) of mixed sequences, or (c) the sequence was of high quality, but matched a non‐bird taxon (often earthworms and moths). Ambiguous samples were excluded when identifying informative biomarkers in the rat GI microbiome (see *biomarker and microbiome matching* subsection below).

We verified that the nine bird species found in the study sites (Table S1) had sequences in GenBank for either the COI gene, the Cytb gene, or both by custom searches. Two bird species (*C. sandwichensis*, *L. leucomelanos*) did not have reference sequences available for COI, but the *Lophura* genus had reference sequences. All bird species had reference sequences for the Cytb gene. In the ‘consumed bird’ samples, we did not find evidence of multiple sequencing (i.e., superposition of several sequences from different species in the same sample).

### 2.5 Microbiomes of rat feces and rat stomachs (rat GI microbiome)

We conducted Illumina high‐throughput sequencing of 16S rRNA gene amplicons to characterize bacterial communities in rat feces and rat stomachs. We amplified DNA using the same primers as used for the chicken samples (515F, 939R). We used a two‐step library preparation to generate 16S rRNA amplicons for sequencing. First, we performed duplicate PCR reactions for each sample, and included negative extraction controls and negative PCR controls. Each 25‐μl amplicon PCR reaction consisted of 1.25 U of AmpliTaq Gold DNA Polymerase (ThermoFisher), 1.5 μM MgCl, 200 nM dNTPs, 500 nM reverse primer with overhang adapter, 500 nM forward primer with overhang adapter, 20 ng/μl BSA and 3 μl DNA template. PCR conditions were 95°C for 7 m, followed by 30 cycles of 95°C for 45 s, 55°C for 30 s, 72°C for 45 s and a final extension (72°C for 7 m). Then, we performed index PCR, adding custom i5 and i7 adaptors to the PCR amplicons to uniquely identify each sample. Each 50‐μl index PCR reaction consisted of 25 μl of 2× Phusion Hot Start II HF Master Mix, 1 mM i5 primer, 1 mM i7 primer, and 5 μl amplicon PCR DNA template. PCR conditions were 98°C for 2 m, followed by eight cycles of 98°C for 20 s, 62°C for 30 s, 72°C for 30 s and a final extension (72°C for 2 m). Between each PCR reaction, PCR products were cleaned with Speed‐beads (Rohland & Reich, 2012). We quantified samples using a Qubit Fluorometer (Invitrogen), and then pooled them equimolar to make a final library pool. We conducted size selection for the target band using E‐Gel EX 2% agarose (Invitrogen) and Qiagen QIAquick Gel Extraction kit. We conducted Illumina MiSeq high‐throughput sequencing on one sequencing run (v3 chemistry: 2 × 300 bp kit) to characterize the bacterial communities of each sample.

We quality filtered and processed sequence reads using the program Quantitative Insights Into Microbial Ecology 2 (vQIIME2‐2018.8; Bolyen et al., 2019). We imported demultiplexed reads from the Illumina MiSeq and filtered them using the following criteria: ‐‐p‐trunc‐len‐f 270 ‐‐p‐trunc‐len‐r 200 ‐‐p‐trim‐left‐f 19 ‐‐p‐trim‐left‐r 23. Sequences were then categorized into ASVs via the dada2 pipeline (Callahan et al., 2016) and taxonomy was assigned by aligning ASVs with the Greengenes 13_8 99% database (DeSantis et al., 2006) using a Naïve Bayes classifier trained on the 515F/939R region. A phylogenetic tree was built using the fasttree algorithm (Price et al., 2010). These files were imported into R using the package ‘phyloseq’ (McMurdie & Holmes, 2013), where all subsequent analyses were conducted. We filtered the data to only contain ASVs that occurred at least once on at least two samples (i.e., filtered singletons). One fecal sample (P‐173A) had low sequence coverage (1,012 sequences) and was not included in analyses.

### 2.6 Biomarker and microbiome matching: informative bacterial taxa of bird consumption and decay status

We compared the sequences of our bacterial biomarker to the sequences of bacteria detected in rat fecal and stomach microbiomes to identify which bacteria were both biomarkers and also present in the GI tract of rats. We used a custom blast analysis in Geneious 8.1 to query our biomarker sequences against the microbiome sequences (Muletz‐Wolz et al., 2017). We used a megablast program, having Geneious return results as query‐centered alignment data only and returning only the top hit. We considered a rat GI microbiome ASV as a match to a biomarker ASV if they matched at ≥97% sequence similarity. We caution that this does not necessarily mean these bacteria are the same species or strains, only that they are strong candidates particularly at ≥99% sequence similarity (which a majority of our biomarkers are—see in results Table 2).

We identified which bacterial biomarkers were only detected in rat samples that showed evidence of bird consumption. We did so to eliminate any potential biomarkers that may be part of the resident GI microbiome (i.e., present in rats that did not consume birds), and therefore uninformative. We subset our rat fecal and stomach microbiome data to (a) only contain ASVs that matched bacterial biomarkers, and (b) only contain rats that we had unambiguously determined to have consumed bird (*n* = 20) or to have not consumed bird (*n* = 11) from diet analysis (see *diet analysis of rat feces and rat stomachs* above). We exported a site (rat) by species (ASV) matrix from R of this subset data, and manually identified which biomarkers were only present in rat samples that showed evidence of bird consumption. We only considered biomarkers as informative when they were present in rats that consumed bird and absent from rats that did not consume bird.

### 2.7 Statistical analyses

All statistical analyses were performed in R version 3.5.3 (R‐Core‐Team, 2019). In the rat microbiome dataset, we quantified microbiome structure with (a) two measures of alpha diversity (ASV richness and Faith's phylogenetic diversity [Faith's PD]), (b) three measures of beta diversity (Jaccard, unweighted UniFrac, and Bray–Curtis), and (c) two measures of bacterial relative abundance using sequence counts (two taxonomic levels: bacterial ASV and bacterial phylum). Prior to conducting Bray–Curtis analyses, we performed proportion normalization on the raw sequence counts (i.e., total sum scaling) to correct for biases associated with unequal sequencing depth on this abundance‐weighted measures (McMurdie & Holmes, 2014; Weiss et al., 2017). Variation in sequencing depth was approximately 16x. Therefore, we included sequence count as an additional explanatory variable in our alpha and beta diversity analyses (Weiss et al., 2017) to account for variation in sequencing depth among samples and to avoid statistical issues associated with sequence count rarefaction (McMurdie & Holmes, 2014). If sequencing depth was significant, we performed analyses on a rarefied dataset to verify that sequencing depth was not driving the significance of the biological effect. We found that sequencing depth did not affect our statistical results or biological inference, and therefore we only report the statistics for the raw sequence data (non‐rarefied) in the results. Our sequencing depth provided adequate sampling of the community (Figure S1).

We analyzed rat stomach versus rat fecal samples to determine how the two sample types varied in microbiome structure. To determine if alpha diversity differed between sample types, we conducted ANOVAs with ASV richness or Faith's PD as the response variable and rat sample type as the explanatory variable. We used the *ANOVA* function in the ‘car’ package with type II sums of squares to determine significance. To determine if bacterial community composition (beta diversity) differed between sample type, we conducted PERMANOVAs with Jaccard, unweighted UniFrac or Bray–Curtis distance as the response variable and rat sample type as the explanatory variable. We verified that beta dispersion was similar between sample types and not influencing the PERMANOVA results by conducting a PERMDISP. To determine if bacterial relative abundance differed between sample types, we used the package ‘DAtest’ to first rank various statistical methods used to test for differential abundance (Russel et al., 2018). We filtered low abundance ASVs (present in <7 samples) using the function *preDA*. Then, we input the raw sequence counts of the filtered ASV table (or phylum level table with no filtering) and used the function *testDA* to allow each statistical method to perform its default transformation on the data. We used the top two differential abundance tests that had the lowest False Positive Rate (Russel et al., 2018) in our statistical analyses with bacterial abundance at two taxonomic levels (bacterial ASV or bacterial phylum) as the response variable and rat sample type as the explanatory variable. We report only ASVs or phyla that were significant (after being corrected for multiple comparisons) in both of the top ranked differential abundance tests (in our analyses = quasi‐poisson generalized linear model [function *DA.qpo*] and Welch *t*‐test [function *DA.ttt*]).

We determined if microbiome structure could predict whether a rat consumed a bird. We subset our rat GI microbiome dataset to only include rats that we had unambiguously determined to have consumed bird (*n* = 20) or to have not consumed bird (*n* = 11) from diet analysis. To determine if ASV richness or Faith's PD was predictive of bird consumption, we used a generalized linear model with a binomial distribution with bird in diet (yes or no) as the response variable and the alpha diversity metric (ASV richness or Faith's PD), sample type and their interaction as response variables. For beta diversity, we were unable to find a statistical test that can use a distance matrix as an explanatory variable. Therefore, we determined if bacterial community composition (beta diversity response variable: Jaccard, unweighted UniFrac or Bray–Curtis distance) differed between rats that consumed birds and those that did not and if sample type mattered (bird in diet, sample type, and their interaction as explanatory variables). Finally, we conducted indicator species analysis to see if particular bacterial ASVs or particular bacterial genera (table merged at the genus level) could differentiate rat samples based on bird consumption. We filtered the ASV table to only contain bacterial taxa found in at least 15% of samples to reduce the number of comparisons and likelihood for false positives (ASVs *n* = 124). We used the *multipatt* function in the ‘indicspecies’ package (De Caceres & Legendre, 2009) on a presence‐absence matrix. We corrected for multiple comparisons using false discovery rate corrections. We considered an ASV as an indicator species if it had a *p* < .05 and an indicator stat value of more than 0.7 (as in Becker et al., 2015; Castro‐Luna et al., 2007). An indicator value of 1 indicates that the ASV was observed in all the samples from one group and completely absent from the other group, while an indicator value of 0 indicates that the ASV was widely distributed across both groups.

## 3 RESULTS

### 3.1 Identification of bacterial biomarkers from decaying chicken tissue

We generated 215,369 high‐quality sequences from 66 chicken tissue samples. Average sequencing depth per biological sample was 3,193 sequences (min = 5, max = 12,338). Many of the early sampling days (Day 0 lab, Day 0 field, Day 1) had low sequence coverage likely due to few microbes colonizing the freshly bleached tissue. We identified 79 ASVs present on chicken tissue belonging to three bacterial phyla (Proteobacteria, Bacteroidetes, Firmicutes), with 99% of sequences belonging to the Proteobacteria phylum. The majority of bacterial biomarker ASVs belonged to the genus *Pseudomonas* within the phylum Proteobacteria (Figure S2, 87% of sequences).

**FIGURE 3:**
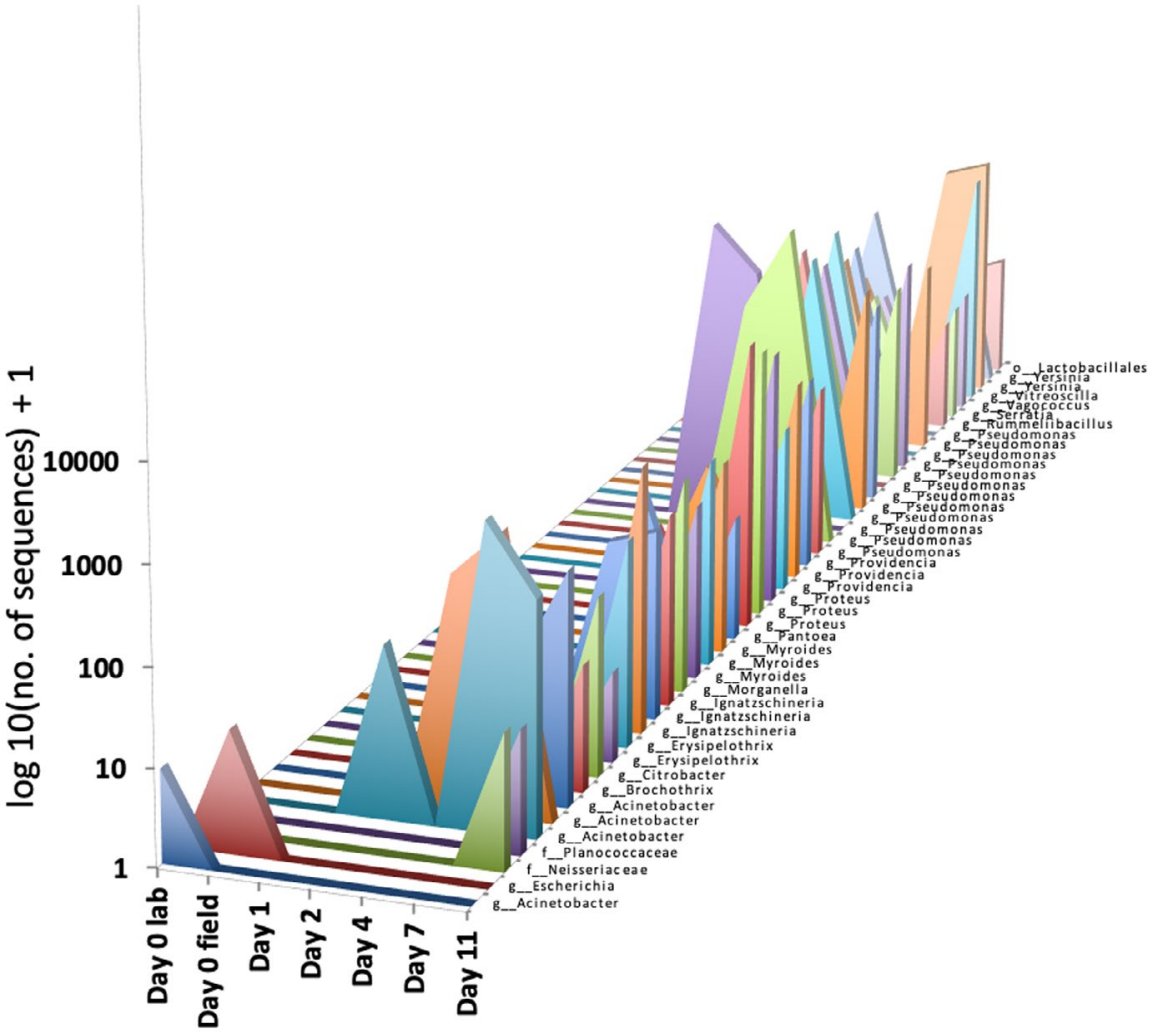
Biomarkers identified from decaying chicken tissue sampled over 11 days. We identified two bacterial ASVs as fresh tissue bacterial biomarkers and 42 ASVs as decayed tissue bacterial biomarkers

We found similar microbiome composition on decomposing chicken tissue among the ten replicate sites (Jaccard PERMANOVA: *p* = .291; Bray–Curtis: *p* = .37), but differences across sampling days (Figure S2). Site replicates were pooled together to identify fresh tissue biomarkers (present in only Day 0 lab, Day 0 field, and/or Day 1) and decayed tissue biomarkers (present only in Days 2, 4, 7, and/or 11). We identified 44 ASVs of the 70 chicken‐tissue‐associated ASVs as biomarkers. Two ASVs we identified as fresh tissue bacterial biomarkers, and 42 ASVs we identified as decayed tissue bacterial biomarkers (Figure 3).

### 3.2 Diet analysis to determine if rats consumed bird

We found that the rat samples we collected included rats that had consumed birds recently and rats that had not consumed bird recently (Table 1). We identified 20 rat samples that contained bird DNA (*n* = 7 feces, *n* = 13 stomachs). We identified 11 rat samples that contained no bird DNA (*n* = 9 feces, *n* = 2 stomachs). We had 20 samples that we could not unambiguously confirm the presence or absence of bird DNA; we considered these samples as ambiguous, and excluded these samples from when we were identifying the putative biomarkers. All sequences and their associated data are provided at https://doi.org/10.6084/m9.figshare.13274816.

**TABLE 1:** Detection of bird DNA in rat samples

Sample type	Bird in diet	Sample size (*n*)
feces	Ambiguous	12
feces	No	9
feces	Yes	7
stomach	Ambiguous	8
stomach	No	2
stomach	Yes	13
	Total =	51

Note

Fecal and stomach samples were collected from different rats.

### 3.3 Characterization of rat GI microbiome

**FIGURE 4:**
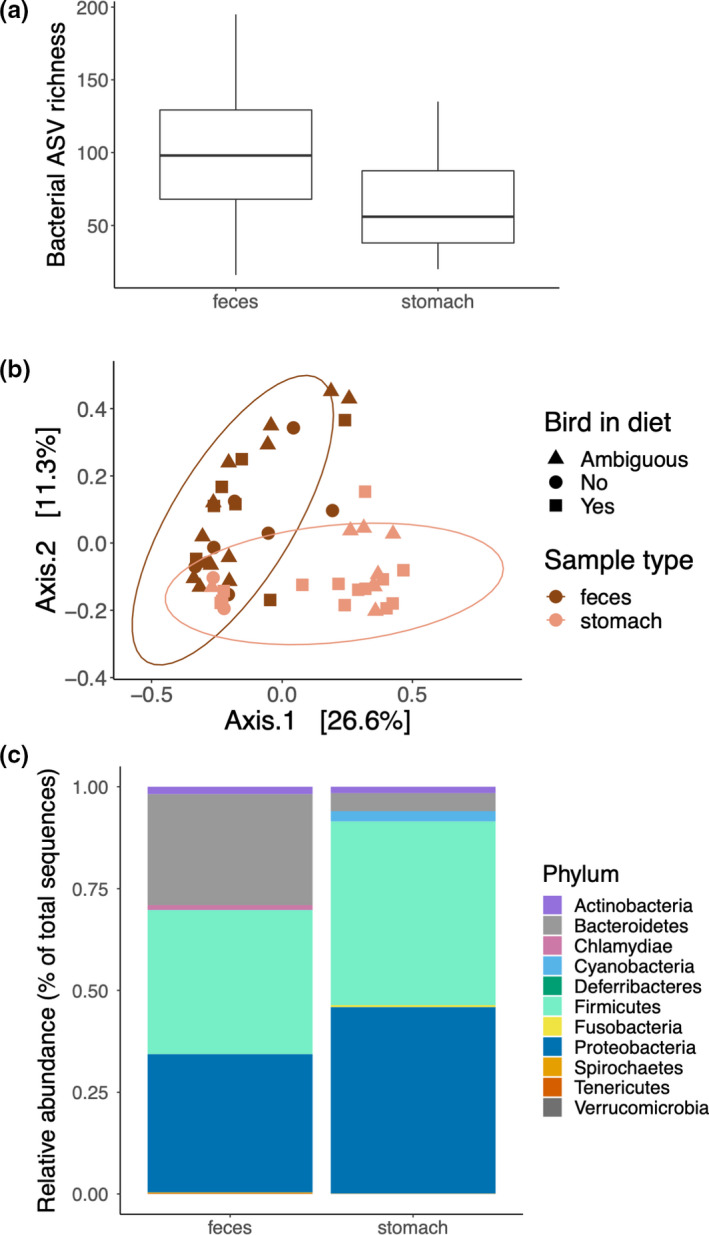
Microbiome structure by rat sample type. Fecal samples had (a) higher bacterial ASV richness and (b) dissimilar community composition (UniFrac distances shown here with 95% confidence ellipses: shapes indicative of bird in diet status) from stomach samples. The relative abundance of bacterial phyla was generally similar (c), except Bacteroidetes was higher in feces compared to stomach samples

We generated 1,230,163 high‐quality sequences from 51 rat samples (*n* = 28 fecal samples, *n* = 23 stomach samples). Average sequencing depth per biological sample was 24,121 sequences (min = 4,762, max = 74,921). We identified 987 ASVs present in the rat GI tract belonging to 11 bacterial phyla (Figure 4c). The majority of ASVs belonged to the phyla Proteobacteria, Firmicutes and Bacteroidetes. Stomach and fecal samples cumulatively had 987 bacterial ASVs, with 407 ASVs detected in both sample types. The shared ASVs made up 84.5% of the sequences from stomach samples and 67.7% of sequences from fecal samples, indicating that many ASVs were shared between sample types.

Microbiome structure generally differed between rat sample type. Rat fecal samples had higher bacterial ASV richness (Figure 4a, ANOVA: *F*_1,49_ = 9.82, *p* = .003) and higher phylogenetic diversity (Faith's PD ANOVA: *F*_1,49_ = 7.02, *p* = .011) than rat stomach samples. Rat feces and rat stomachs differed in bacterial community composition (Figure 4b, Jaccard PERMANOVA: Pseudo‐ *F*_1,49_ = 4.30, *R* ^2^ = 8%, *p* = .001; UniFrac: Pseudo‐ *F*_1,49_ = 7.69, *R* ^2^ = 14%, *p* = .001; Bray–Curtis: Pseudo‐ *F*_1,49_ = 2.85, *R* ^2^ = 6%, *p* = .001), but had similar dispersion within their respective communities (Jaccard PERMDISP: *p* = .2, UniFrac: *p* = .7, Bray–Curtis: *p* = .1). Bacterial relative abundances were similar between fecal and stomach samples at the ASV and phylum level, except the phylum Bacteriodetes was higher in rat fecal samples than in stomach samples (Figure 4c, Welch *t*‐test *p* = .007, log2FC −1.72; Quasi‐poisson GLM *p* = .006, log2FC −2.62). There was variation in microbiome structure across kīpuka, but the pattern of higher bacterial ASV richness and phylogenetic diversity, distinct bacterial community composition, and higher Bacteriodetes in fecal versus stomach samples was generally maintained across the kīpuka within each sample type (Figure S3).

### 3.4 Informative bacterial taxa of bird consumption and decay status

We determined if the bacterial biomarkers that we identified from decaying chicken tissue (Figure 3) were able to predict bird consumption and decay status. We detected several decayed tissue bacterial biomarkers in rat GI samples that had consumed bird, but did not detect any fresh tissue bacterial biomarkers. We detected 40 bacterial ASVs in the fecal and stomach microbiomes of rats that matched 22 decayed tissue bacterial biomarkers (matched ≥97% sequence similarity). We then eliminated the bacterial ASVs that were present in rat microbiome samples of rats that did not consume birds. In other words, we eliminated any bacterial biomarker that may have been resident microbial taxa in GI tract of rats. After this filtering step, we had 15 informative bacterial ASVs, which matched seven decayed tissue biomarkers and were found only in rats that had consumed bird (Table 2). Of the 20 rats we confirmed consumed birds through diet analysis, we identified nine rats that potentially consumed birds as carrion (3/7 fecal samples; 6/13 stomach samples) with our 15 informative bacterial ASVs (Table 2). Combining diet and biomarker analysis, we found that rats consumed four different bird species, including one native and three introduced birds, and likely consumed three of those bird species through scavenging (Table 3).

**TABLE 2:** Bacterial ASVs detected on decaying chicken tissue that matched bacterial ASVs in GI microbiomes of rats that consumed birds

Decayed chicken tissue biomarker ASV ID	No. tissue replicates detected in	Rat GI ASV ID	Phylum	Family	Genus	Species	Similarity to decayed tissue biomarker (%)	No. rats detected as consuming bird as carrion
ASV100 (Day 11)	3	e15086c6f06b9ab2db9544a6573e930d	Proteobacteria	Pseudomonadaceae	*Pseudomonas*	*viridiflava*	100	1
		93ae0cb4f1180c5bc85da5a5ad99d4a2	Proteobacteria	Pseudomonadaceae	*Pseudomonas*	*viridiflava*	99	3^#^
ASV103 (Day 11)	2	bd298d360cf7e61a2e1d2abe51467853	Proteobacteria	Enterobacteriaceae	*—*	*—*	99	3^#^
		9e7246ddc0eae83c1a74a316fe72f2f1	Proteobacteria	Enterobacteriaceae	*—*	*—*	99	2^#^
		910acad922c911e9b2270f96326b9bb1	Proteobacteria	Enterobacteriaceae	*—*	*—*	99	1
		12e77d709989ae4da784b662fce24432	Proteobacteria	Enterobacteriaceae	*—*	*—*	99	1^+^
ASV135 (Day 4)	2	f00f91d78f49cfbe68e6554f920ae5d8	Proteobacteria	Enterobacteriaceae	*—*	*—*	99	3^#^
		e15e067244d294ff4e0a4bb7f3149dc9	Proteobacteria	Enterobacteriaceae	*—*	*—*	100	1
		813e7aaa81207e1cf91364ee5691a605	Proteobacteria	Enterobacteriaceae	*—*	*—*	98	3^#^
ASV142 (Day 4)	2	*df*8da8cdf673547d89adba6a22f7d2ee	Proteobacteria	Enterobacteriaceae	*Ewingella*	*americana*	98	3*^#^
		239c51edf1db3559615306d8952fe27e	Proteobacteria	Enterobacteriaceae	*—*	*—*	98	1
ASV147 (Day 4)	2	36d75a97bbb0e49db0d56538ce946b2a	Firmicutes	Carnobacteriaceae	*Carnobacterium*	*NA*	97	1
ASV54 (Day 4)	3	9cd5c63214c4582ee6d84e0bfd903c71	Proteobacteria	Pseudomonadaceae	*Pseudomonas*	*NA*	99	3*^#^
		21301da854c631af0c945541ce91efb8	Proteobacteria	Pseudomonadaceae	*Pseudomonas*	*viridiflava*	98	1
ASV73 (Day 11)	2	868c3e66c9baedb1bf2c6b9328abf35c	Proteobacteria	Enterobacteriaceae	*Morganella*	*morganii*	100	2^#^
							Total no. unique rats =	9

Note

Day in parentheses is the first sampling time point since start of decomposition that biomarker was detected on the chicken tissues. Bacterial biomarkers that were detected in both rat stomachs and rat fecal samples are denoted by an asterisk (*), those only detected in rat feces are denoted by plus sign (^+^), and those detected in multiple kīpuka are denoted by a hash mark (^#^).

**TABLE 3:** Results of Sanger sequence assignment to bird species based on NCBI blast and results of detection of informative bacterial biomarkers of carrion consumption in rat GI microbiome

Primer pair	Sample ID	Sample type	Sequence result	Likely consumed as carrion
BirdF1/AwCintR2 (COI gene region)	P‐165A	feces	Lophura leucomelanos*	
	R‐12‐619	stomach	*Zosterops japonicus*	Yes
	R‐12‐620	stomach	*Zosterops japonicus*	
	R‐12‐623	stomach	*Myadestes obscurus*	
	R‐12‐624	stomach	*Zosterops japonicus*	
	R‐12‐626	stomach	Lophura leucomelanos*	
	R‐12‐627	stomach	*Zosterops japonicus*	
	R‐12‐631	stomach	*Zosterops japonicus*	
	R‐12‐637	stomach	*Zosterops japonicus*	Yes
	R‐12‐640	stomach	*Zosterops japonicus*	
	R‐12‐642	stomach	*Zosterops japonicus*	Yes
	R‐12‐661	stomach	*Zosterops japonicus*	Yes
	R‐12‐662	stomach	*Zosterops japonicus*	Yes
	R‐12‐665	stomach	*Zosterops japonicus*	Yes
CytbCorL/CytbCor3 (Cytb gene region)	P‐002A	feces	*Meleagris gallopavo*	Yes
	P‐003A	feces	*Meleagris gallopavo*	
	P‐018A	feces	*Myadestes obscurus*	Yes
	P‐023A	feces	*Myadestes obscurus*	Yes
	P‐025A	feces	*Myadestes obscurus*	
	P‐166A	feces	*Myadestes obscurus*	
	R‐12‐619	stomach	*Zosterops japonicus*	Yes
	R‐12‐623	stomach	*Myadestes obscurus*	
	R‐12‐626	stomach	Lophura leucomelanos*	
	R‐12‐627	stomach	*Zosterops japonicus*	
	R‐12‐631	stomach	*Zosterops japonicus*	
	R‐12‐637	stomach	*Zosterops japonicus*	Yes

Note

Asterisk denotes that sequences matched to *Lophura nycthemera* (not found in Hawaii) and was considered to indicate rat consumption of the *Lophura* species present in our study sites (*Lophura leucomelanos*), which does not have a reference COI sequence in GenBank.

As part of an exploratory analysis, we used the informative bacterial ASVs for decayed tissue consumption to suggest the bird consumption status for some of the ‘ambiguous’ rat samples from diet analysis. We had 20 rat samples (12 fecal and eight stomach samples) that we originally assigned as ambiguous for bird consumption through diet analysis. We suggest that five of those 20 rats (three fecal and two stomach samples) may have consumed birds as carrion; those rat samples contained at least two of the 15 informative bacterial ASVs for decayed bird tissue in their microbiome.

### 3.5 Likelihood rat GI microbiome structure can predict bird consumption

We also tested if diversity measures of rat GI microbiome structure could predict bird composition. Rat GI microbiome structure did not predict bird consumption (alpha diversity GLMs: ASV richness *p* = .3, Faith's PD *p* = .3: beta diversity PERMANOVAs: Jaccard *p* = .2, UniFrac *p* = .2, Bray–Curtis *p* = .5). We detected one ASV, *Prevotella copri*, that was predominately found in rats that did not consume birds (7/11 rats) while generally absent from rats that had consumed bird (2/20 rats; Indicator Species Analysis *p* = .041, stat value = 0.74).

## 4 DISCUSSION

Scavenging is widespread and significant in most food webs, but is often significantly underestimated, producing inflated predation rates and underestimated indirect effects (Wilson & Wolkovich, 2011). We used a multi‐pronged approach of new molecular and classic ecological methods to document bird scavenging by invasive rats in Hawaii. We showed that bacterial biomarkers, linked to the successional stage of bird carcass decay, likely persist through digestion as biomarkers of carrion consumption in both rat stomachs and fecal material. Our forensic microbiology tool provides vital information to identify species in networks, detect cryptic linkages that occur beyond our observation (e.g., at night, in the forest canopy, in burrows or crevices) and can be used to help build weighted network models with predictions for food web stability (Deng et al., 2012; Evans et al., 2016; Traugott et al., 2013).

We found that 39% of the rats we sampled (20/51) unambiguously consumed birds, and nearly half of those—18% in total (9/51)—may have consumed birds as carrion by the presence of bacterial biomarkers in their GI microbiome. It is possible that the other 11 rats that we confirmed consumed bird may have consumed the birds as prey given the absence of decayed tissue biomarkers. We found that four of the nine birds repeatedly detected in our study area were detected in the diet of rats, which included one native bird species (*M. obscurus*) and three introduced bird species (*L. leucomelanos*, *M. gallopavo*, *Z. japonicus*). Two of the native bird species did not have reference sequences for our COI primer set, which may have reduced our likelihood to detect them; however, we did not detect these as diet items with the Cytb primer set for which there were reference sequences in GenBank. The Kalij pheasant (*L. nycthemera*) was the only species lacking biomarkers attributable to scavenging, while the other three species had individuals that may have been consumed through scavenging.

The rats that we identified as likely consuming birds through scavenging contained at least one of 15 informative bacterial ASVs of carrion consumption in their GI microbiome. We caution that these biomarkers may be generally indicative of other vertebrate carrion consumption. In our study system, there are few other vertebrate diet items, but we cannot rule out the possibility that those were also consumed as carrion. In future work, if diet samples are analyzed more broadly, for example using a metabarcoding approach (Forin‐Wiart et al., 2018), and only bird species are identified, then identified bacterial biomarkers in the feces or guts most likely originated from the bird in the diet and not some other vertebrate. Additionally, future work should determine if bacterial biomarkers can be considered specific to a particular diet group (e.g., birds or mammals or reptiles) by conducting feeding trials (Zarzoso‐Lacoste et al., 2011).

Microbial composition in the environment and on animals can often differ across the landscape (Bisson et al., 2009; Muletz Wolz et al., 2018). Across the kīpuka we sampled, we found that eight of the 15 informative bacterial biomarkers occurred in rats sampled in multiple kīpuka indicating reproducibility of bacterial signatures of decay within our study area. From studies of human and mouse carcass decomposition, evidence suggests that microbial signatures of decay are often predictable on a larger scale across soil types, seasons and host species (Belk et al., 2018; Metcalf et al., 2016). Occurrence of a specific subset of biomarkers from a microbial community offers the potential to develop cheaper, targeted assays (Yan et al., 2015). Replicating our study across a larger geographic region to determine if similar bacterial carrion biomarkers are identified would provide support for developing more targeted assays.

We identified bacterial biomarkers of decayed tissue (i.e., carrion) consumption that we considered indicative of scavenging, but were unable to identify bacterial biomarkers of fresh tissue that could have been indicative of predation. In our system, it is most likely that predation with immediate consumption and scavenging are the primary feeding strategies as rats are the primary consumers of bird nestlings and fledglings in the kīpuka. However, in other systems where rats may consume large prey items over a series of days, it would be important to differentiate predation and consumption over several days from scavenging. We suggest that for future studies to conclusively differentiate (a) predation and immediate consumption, (b) predation and consumption over several days, and (c) scavenging, it will require direct observation or manipulation to identify and validate biomarkers for each category. In our study, we did find two bacterial ASVs in the decaying chicken tissue study indicative of fresh tissue (and potentially predation), but we did not detect those bacterial ASVs in rat GI microbiomes. The chicken tissue was sterilized with bleach prior to placing it in the field in order to remove any bacteria that accrued on the surface of the meat during its processing for sale. This may have impacted our ability to identify biomarkers of fresh bird tissue, and few bacterial taxa were present at early sampling time points. Likewise, the microbiome of commercially produced chicken is unlikely to resemble that of wild forest birds at initial time points, but during decomposition we expected that the decaying chicken microbiome would be representative of a general bird decomposition microbiome (Belk et al., 2018; Metcalf et al., 2016). We chose to use store‐bought, antibiotic‐free chicken as a proof of concept and to determine if using birds in Hawaii in a decomposition study would be warranted in the future, as many are vulnerable or endangered.

In this study, we determined that invasive rats in Hawaii consumed tissues of both native and introduced birds as diet items. One of the birds identified was the Japanese white‐eye (*Z. japonicus*), an invasive bird that since its introduction in 1929 has proliferated to become the most abundant bird species on the islands (Berger, 1981; Van Riper, 2000). In future studies, we suggest using whole *Z. japonicus* carcasses in decomposition and/or feeding trials as they are invasive, abundant, and a known rat diet item. It would be pertinent to compare future studies using wild birds with the bacterial biomarkers identified in our chicken decomposition study to verify how the impact of whole unaltered carcasses impact biomarker discovery.

The bacterial biomarkers we identified have the potential to be used to determine if rats had consumed birds or not, in cases where the results from diet analysis are ambiguous. However, with our currently limited knowledge on the specificity of bacterial biomarkers of decay to particular diet items, we provide an exploratory analysis with potential for use in future work. We had a relatively high number of ambiguous samples, and this number may have been reduced with more replication of PCR and sequencing (e.g., six PCR replicates per primer pair such as in Zarzoso‐Lacoste et al., 2016). However, we still would anticipate some ambiguity even with high laboratory replication and sequencing (Forin‐Wiart et al., 2018; Zarzoso‐Lacoste et al., 2016). Nonetheless, we were able to robustly identify 20 rats as ‘consumed bird’ and 11 rats as ‘not consumed birds’ for analyses and identification of biomarkers, which was one of our primary goals of the research.

Molecular diet analysis of fecal material is gaining traction among molecular ecologists as a noninvasive method to study diet and trophic interactions, particularly for invasive species (Egeter et al., 2019; Zarzoso‐Lacoste et al., 2016). However, molecular techniques in diet analysis are not without issue, and some ambiguity can exist such as repeatability in food item detection (Forin‐Wiart et al., 2018) and PCR bias (Nichols et al., 2018). Integrating diet analysis with forensic microbiology can provide a suite of tools to better inform conclusions on food webs, including resolving ambiguities in diet analysis and elucidating whether diet items were likely consumed through predation versus scavenging.

In order to validate that rat fecal samples would provide similar biological conclusions as stomach samples, we utilized rat stomach and fecal samples from 18 different kīpuka that were collected as part of a larger study in which rat removals were performed and individuals were ethically euthanized (stomachs) or traps were placed in control non‐rat removal plots (feces). We found that while rat stomachs and rat feces differed in their microbiome structure with reproducibility in those differences across kīpuka, two of 15 bacterial biomarkers were detected in both sample types (*Ewingella americana* and a *Pseudomonas* sp.). In paired samples from individual animals of other vertebrate species, fecal samples differed from stomach samples in microbiome structure, including in bats (Ingala et al., 2018) and birds (Drovetski et al., 2018; Videvall et al., 2018). Ingala et al. (2018) proposed that fecal microbiomes may be more species rich than stomachs, because bacterial DNA from diet items can be retained in pockets of undigested material in feces. Recently produced fecal material may serve as a better sample type than stomachs for bacterial biomarkers of conditions prior to consumption, given the greater potential for retention of bacterial DNA from those diet items in feces. Likewise, collecting fecal material is less time‐intensive and more humane than stomachs.

Genomic forensics have many applications to wildlife conservation settings and to food web ecology. Rats (*Rattus* spp.) have been introduced to nearly 90% of the world's islands and are among the most detrimental invaders (Harper & Bunbury, 2015; Martin et al., 2000; Shiels et al., 2014). Knowing the magnitude of predation by invasive rats can inform management practices. Yet, monitoring large numbers of bird nests in the forest canopy to determine if rats caused significant nest failure through predation can be time‐ and cost‐prohibitive. For instance, extensive previous monitoring of bird nests identified a general cause of nest failure (i.e., “presumed predation”) in only ~15% of cases (Knowlton, unpublished data). Similarly, observing foraging behavior to assess rat diets becomes impractical with increasing canopy or cliff height, and a massive effort is required to obtain the statistical power to detect subtle population and community impacts. Repeated temporal snapshots are needed to observe dietary changes that might result from behavioral shifts in foraging, but invasive techniques such as stomach sampling remove animals from the population and is impractical at a large scale. Our paired diet and microbial forensics approach using noninvasive fecal samples could be implemented as a means to screen larger geographic regions for rat predation versus scavenging; certain areas could then be identified for validation with direct observation. Direct observations would be informative to validate bacterial biomarkers that could conclusively differentiate predation and immediate consumption from predation and consumption over several days and from scavenging.

Nest predation by black rats in Hawaii can vary across the landscape from high to extremely low (e.g., 87% [Stone et al., 1984] to 4% [Amarasekare, 1993]). Although dead biomass can subsidize predator populations, it does not contribute demographically to the dynamics of prey populations (Wolkovich et al., 2014). However, the availability of alternative resources, as live prey or as carrion, may be equally influential in supporting higher numerical response or population densities of invasive omnivores impacting populations of conservation concern (David et al., 2017; Grendelmeier et al., 2018; Holt & Bonsall, 2017). Thus, determining the degree of scavenging versus predation is key to determining the impact of invasive rats on bird populations. Our paired diet and microbial forensics approach could be used to examine regional patterns of rat predation versus scavenging on native and introduced bird species, where particular focus would be on identifying localities where rats or other invasive omnivores are shown to be more likely to be consuming native birds through predation. Rat removal is extremely time and labor intensive; therefore, being able to target rat populations that are having direct impacts on native Hawaiian avifauna can be integral to their conservation. Specifically, researchers could collect fecal material from a variety of localities with nonlethal sampling or trapping. Our molecular‐based approaches could then be used to detect bird DNA in diet and look for the informative bacterial biomarkers of predation or carrion consumption, as identified here (carrion consumption biomarkers only) or in future research. Specific areas could then be targeted for validation with on ground observations, and appropriate management decisions could be made. Forensic genomic and microbiology methods applied in wildlife ecology have great potential to detect and quantify species interactions that are remote, rare, and challenging to measure. However, there is a need for basic research, such as the research we present here and those by others (Guo et al., 2016; Pechal & Benbow, 2016), before this can be formalized as a widely accepted tool.

## CONFLICT OF INTEREST

The authors have declared that no competing interests exist.

## AUTHOR CONTRIBUTION

**Carly R. Muletz‐Wolz:** Formal analysis (lead); Investigation (lead); Visualization (lead); Writing‐original draft (lead); Writing‐review & editing (lead). **Erin Wilson Rankin:** Conceptualization (equal); Investigation (equal); Resources (equal); Writing‐review & editing (equal). **Sarah McGrath‐Blaser:** Formal analysis (equal); Investigation (equal); Visualization (equal); Writing‐review & editing (equal). **Madhvi Venkatraman:** Investigation (equal); Resources (equal); Writing‐review & editing (equal). **Jesús E Maldonado:** Conceptualization (equal); Funding acquisition (equal); Resources (equal); Writing‐review & editing (equal). **Daniel S Gruner:** Conceptualization (equal); Funding acquisition (equal); Resources (equal); Supervision (equal); Writing‐review & editing (equal). **Robert C Fleischer:** Conceptualization (equal); Funding acquisition (equal); Resources (equal); Supervision (equal); Writing‐review & editing (equal).

## Supporting information

Supplementary Material

## Data Availability

Demultiplexed high‐throughput sequence data and associated metadata have been deposited in the National Center for Biotechnology Information Sequence Read Archive (www.ncbi.nlm. nih.gov/sra) under BioProject IDs: PRJNA573692 (chicken decomposition study) and PRJNA573693 (rat GI microbiome). All Sanger sequences for diet analysis and associated metadata are provided at https://doi.org/10.6084/m9.figshare.13274816
