# Supplementary material for: Identification of novel bacterial biomarkers to detect bird scavenging by invasive rats
Source: Ecol Evol. 2021 Jan 19;11(4):1814–28. doi: 10.1002/ece3.7171 (PMC7882976; doi:10.1002/ece3.7171)
Supplement: Supplementary file 1 — Supplementary Material [file ECE3-11-1814-s001.docx]

**Supporting information for:**

**‘Validation of novel bacterial biomarkers to identify bird scavenging by invasive rats’**

Carly R. Muletz-Wolz, Erin Wilson Rankin, Sarah McGrath-Blaser, Madhvi Venkatraman, Jesús E. Maldonado, Dan S. Gruner, Robert C. Fleischer

*To whom correspondence should be addressed. E-mail: muletzc@si.edu

This file includes:

Table S1. Birds repeatedly detected in the study area kīpuka

Table S2. Locations of the chicken tissue samples placed in the field.

Table S3. Number of rat samples collected from each kīpuka per sample type.

Figure S1. Rarefaction plot of rat gastrointestinal (GI) microbiome samples.

Figure S2. Relative abundance of bacterial genera over time on chicken tissue samples.

Figure S3. Rat GI microbiome structure by kīpuka.

**Table S1**. Birds that are repeatedly detected in the study area kīpuka.

| **Origin** | **Common name** | **Genus** | **Species** |
| --- | --- | --- | --- |
| Native | Elepaio | *Chasiempis* | *sandwichensis* |
| Native | Oma'o | *Myadestes* | *obscurus* |
| Native | 'Apapane | *Himatione* | *sanguinea* |
| Native | 'I'iwi | *Drepanis* | *coccinea* |
| Native | Hawaii 'Amakihi | *Chlorodrepanis* | *virens* |
| Introduced | Kalij Pheasant | *Lophura* | *leucomelanos* |
| Introduced | Japanese White-eye | *Zosterops* | *japonicus* |
| Introduced | Red-billed Leiothrix | *Leiothrix* | *lutea* |
| Introduced | Wild turkey | *Meleagris* | *gallopavo* |

**Table S2.** Locations of the tissue samples in the field chicken decomposition study, placed in kīpuka 18 and 19.

| **Location Name** | **Latitude** | **Longitude** | **Altitude (m)** | **Distance (km) from Location 1** |
| --- | --- | --- | --- | --- |
| 1 | 19.674163 | -155.331587 | 1497 | 0 |
| 2 | 19.674286 | -155.331543 | 1509 | 0.014 |
| 3 | 19.67436 | -155.331743 | 1523 | 0.037 |
| 4 | 19.674434 | -155.33202 | 1533 | 0.067 |
| 5 | 19.674379 | -155.332255 | 1558 | 0.093 |
| 6 | 19.674452 | -155.333239 | 1560 | 0.196 |
| 7 | 19.674532 | -155.33361 | 1560 | 0.236 |
| 8 | 19.674799 | -155.333569 | 1555 | 0.266 |
| 9 | 19.675122 | -155.333674 | 1551 | 0.304 |
| 10 | 19.675353 | -155.33357 | 1551 | 0.332 |

**Table S3.** Number of rat samples collected from each kīpuka per sample type.

| Sample type | Kipuka ID | Sample size (n) |
| --- | --- | --- |
| feces | 0 | 3 |
| feces | 3 | 2 |
| feces | 4 | 2 |
| feces | 18 | 9 |
| feces | 19 | 3 |
| feces | 20 | 3 |
| feces | 22 | 5 |
| feces | 32 | 1 |
| stomach | 5 | 6 |
| stomach | 6 | 2 |
| stomach | 12 | 1 |
| stomach | 13 | 1 |
| stomach | 14 | 1 |
| stomach | 15 | 4 |
| stomach | 21 | 4 |
| stomach | 23 | 1 |
| stomach | 29 | 2 |
| stomach | 36 | 1 |

**Figure S1.** Rarefaction plot of sequence counts and bacterial ASV richness for rat fecal and stomach samples. We had sufficient sequencing depth to reach a horizontal asymptote for all samples, indicating the ASV richness estimates are good indicators of true bacterial ASV richness.

**Figure S2.** Relative abundance of dominant genera over time on chicken tissue samples.

**Figure S3.** Rat GI microbiome structure by kīpuka. While some kīpuka within each sample type varied in microbiome structure, the kīpuka where fecal samples were collected (0, 3, 4, 18, 19, 20, 22) generally had (a) higher bacterial ASV richness, (b) dissimilar community composition, and (c) higher relative abundance of the bacterial phylum Bacteroidetes compared to kīpuka where stomach samples were collected (5, 6, 15, 21, 29).
